# The Thermal Stability of Janus Monolayers SnXY (X, Y = O, S, Se): Ab-Initio Molecular Dynamics and Beyond

**DOI:** 10.3390/nano12010101

**Published:** 2021-12-29

**Authors:** Yufeng Luo, Shihao Han, Rui Hu, Hongmei Yuan, Wenyan Jiao, Huijun Liu

**Affiliations:** Key Laboratory of Artificial Micro- and Nano-Structures of Ministry of Education and School of Physics and Technology, Wuhan University, Wuhan 430072, China; luoyuf_0902@whu.edu.cn (Y.L.); hansh123@whu.edu.cn (S.H.); ruihu16@whu.edu.cn (R.H.); hmyuan@whu.edu.cn (H.Y.); jiaowy@whu.edu.cn (W.J.)

**Keywords:** thermal stability, Janus monolayer, ab-initio molecular dynamics, atomic displacement parameter

## Abstract

In recent years, the Janus monolayers have attracted tremendous attention due to their unique asymmetric structures and intriguing physical properties. However, the thermal stability of such two-dimensional systems is less known. Using the Janus monolayers SnXY (X, Y = O, S, Se) as a prototypical class of examples, we investigate their structure evolutions by performing ab-initio molecular dynamics (AIMD) simulations at a series of temperatures. It is found that the system with higher thermal stability exhibits a smaller difference in the bond length of Sn–X and Sn–Y, which is consistent with the orders obtained by comparing their electron localization functions (ELFs) and atomic displacement parameters (ADPs). In principle, the different thermal stability of these Janus structures is governed by their distinct anharmonicity. On top of these results, we propose a simple rule to quickly predict the maximum temperature up to which the Janus monolayer can stably exist, where the only input is the ADP calculated by the second-order interatomic force constants rather than time-consuming AIMD simulations at various temperatures. Furthermore, our rule can be generalized to predict the thermal stability of other Janus monolayers and similar structures.

## 1. Introduction

During the past decades, graphene and other two-dimensional (2D) materials have attracted widespread attention from the science community [1,2,3,4,5,6,7]. Among them, the so-called double-faced Janus monolayers are of particular interest since they lack both the in-plane inversion and out-of-plane mirror symmetry. In 2017, the Janus MoSSe was successfully fabricated from 2D transition metal dichalcogenide (TMD) MoS_2_, where the S atoms in the top layer are replaced by the Se through the modified chemical vapor deposition (CVD) method [8]. Since then, there has been a growing interest in such kinds of systems due to their intriguing physical properties, such as intrinsic Rashba-type spin splitting [9], high piezoelectric performance [10], and strong optical absorbance in the visible spectrum [11], as well as higher superconducting critical temperature [12], 2D ferromagnetism with high Curie temperature, and large valley polarization [13], etc. [14].

As various Janus structures have been recently proposed theoretically, it is natural to check their stability before any realistic application. For example, first-principles calculations by Shi et al. [15] demonstrated that the Janus monolayers MXY (M = Ti, Zr, Hf, V, Nb, Ta, Cr, Mo, W and X, Y = S, Se, Te) are mechanically stable. However, the phonon dispersion relations of VXY, TiSTe, and TiSSe exhibit larger imaginary frequencies, indicating that they are dynamically unstable. Inspired by the fabrication of the 2D TMD VSe_2_, Zhang et al. [16] predicted an energetically and dynamically stable Janus structure of VSSe, and further checked its thermal stability at room temperature. Peng et al. [17] proposed a stable Janus monolayer PtSSe, which was later successfully prepared by Sant et al. [18] via the sulfurization of TMD PtSe_2_ under H_2_S atmosphere. Based on two typical Janus systems, MoSSe and BiTeI, Riis-Jensen et al. [19] reported a high-throughput study on 216 derived monolayers, where 93 were identified to be stable according to the calculated formation energies, phonon dispersions, and elastic constants. Using first-principles calculations, Yuan et al. [20] found that the formation energies of Janus MnSTe, MnSeTe, MnSSe, and VSeTe can be comparable to those of the parents MnS_2_, MnSe_2_, MnTe_2_, VSe_2_, and VTe_2_. In addition, previous ab-initio studies confirmed the chemical, dynamical, and mechanical stability of Janus monolayer SnSSe, which exhibits promising optoelectronic properties for solar cells applications and can be also used as appealing industrial waste thermoelectric materials [21,22,23].

Although many Janus monolayers have been proposed to be energetically and dynamically stable in recent years [19,20,21,22,23,24,25,26,27], less is known about their thermal stability and an explicit understanding is therefore quite necessary, especially considering various application potentials at different temperature regions. In this work, using the Janus monolayers SnXY (X, Y = O, S, Se) as a prototypical class of examples, we focus on their thermal stability by performing ab-initio molecular dynamics (AIMD) simulations at a series of temperatures. It is found that the SnSSe is the most stable while the SnOSe is the least, with the SnOS in between. Such obviously different thermal stability is discussed by detailed comparisons of their bond lengths, electron localization functions (ELFs), and atomic displacement parameters (ADPs), all of which are related to the anharmonicity of these Janus monolayers. As it is very time-consuming to perform AIMD simulations at various temperatures, we go further by deriving a simple rule to quickly predict the upper temperatures at which the Janus monolayers SnXY are still thermally stable, and its good transferability is confirmed by several selective examples. We believe that the underlying physics discussed for the SnXY should be similar for other Janus monolayers.

## 2. Computational Methods

The thermal stability of our Janus systems can be evaluated by performing AIMD simulations, which are implemented in the Vienna ab-initio Simulation Package (VASP) [28,29,30]. It should be noted that AIMD combines ab-initio electronic structure calculations and molecular dynamics simulations [31], and such an approach has been successfully used to check the thermal stability of various 2D materials [32,33,34]. The optimized structures were obtained by using the projector augmented wave (PAW) method [35,36] where the exchange-correlation energy was in the form of Perdew–Burke–Ernzerhof (PBE) with the generalized gradient approximation (GGA) [37]. The cutoff energy for the plane-wave basis was set as 600 eV with the residual force on each atom being less than 0.01 eV Å^−1^. 

To eliminate interactions between the Janus monolayer and its periodic images, a vacuum distance of 20 Å along the out-of-plane direction was adopted, as also suggested in the previous study [24]. We used a Monkhorst-Pack ***k***-mesh [38] of 21 × 21 × 1 for sampling the Brillouin zone and the effect of spin-orbit coupling (SOC) was considered in the calculations. The AIMD ran for 10,000 steps with a time step of 1 fs, which was enough to monitor the structure evolutions and evaluate the thermal stability, as generally adopted in many previous works [16,39,40]. Here, we applied a 3 × 3 × 1 supercell and chose a canonical ensemble which ensured the convergence of our results. As the AIMD simulations are very time-consuming, we further proposed that the ADP could be utilized to quickly evaluate the thermal stability of Janus SnXY. By combining the density functional theory (DFT) calculations with the finite displacement method, we could derive the ADP from the second-order interatomic force constants (IFCs) as embedded in the PHONOPY package [41]. In addition, the anharmonicity of the Janus systems was described in terms of the Grüneisen parameter, which can be obtained by solving the phonon Boltzmann transport equation as implemented in the so-called ShengBTE code [42]. The 8 × 8 × 1 and 7 × 7 × 1 supercells were respectively employed for the evaluation of the second- and third-order IFCs, and the twelfth nearest neighbors were considered to ensure convergence of the anharmonic IFCs.

## 3. Results and Discussions

**Figure 1:**
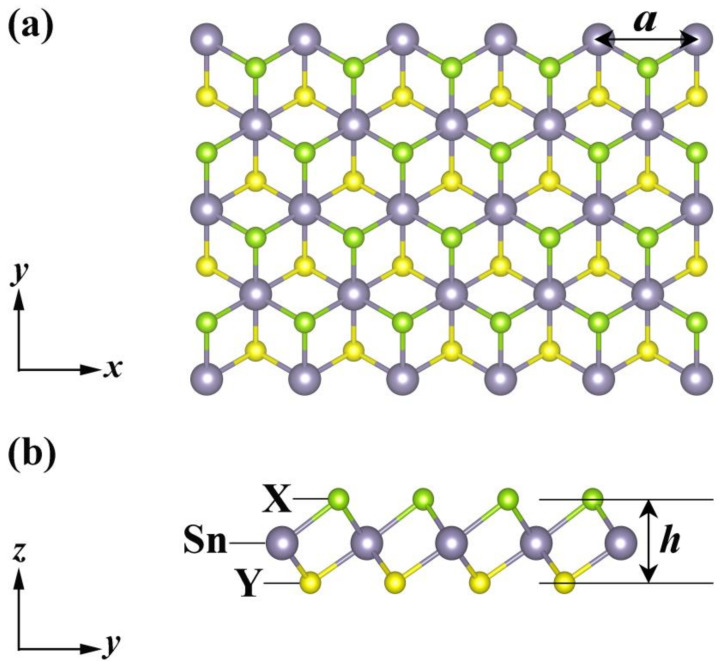
(**a**) Top- and (**b**) side-views of the crystal structure of the Janus monolayers SnXY, where the lattice constant (a) and layer thickness (h) are marked.

Figure 1 illustrates the top- and side-views of the crystal structure of Janus monolayers SnXY (X, Y = O, S, Se), which exhibited a hexagonal lattice and contained three atoms in the primitive cell. Unlike graphene, our systems possessed a sandwiched structure (X−Sn−Y) with the space group of *P*3*m*1, and they lacked both the inversion center and out-of-plane mirror symmetry. As can be found from Table 1, the lattice constant (a), the layer thickness (h), and the bond length ($d_{\mathrm{Sn}–X}$, $d_{\mathrm{Sn}–Y}$) increased with the radii of X and Y atoms, which are in good agreement with those reported previously [21,22,23,24]. If we focus on the difference (Δd) between $d_{\mathrm{Sn}–X}$ and $d_{\mathrm{Sn}–Y}$, we found that Δd was the largest for the Janus SnOSe, smallest for the SnSSe, with the SnOS in between. Such an order just coincided with that of the thermal stability, as will be discussed later. On the other hand, we calculated the phonon dispersion relations of these Janus monolayers (Figure S1 of the Supplementary Material), and the absence of imaginary frequency in the Brillouin zone confirmed that all of them were dynamically stable. Such findings are consistent with those found in previous ab-initio results [21,22,23,24,43].

**Figure 2:**
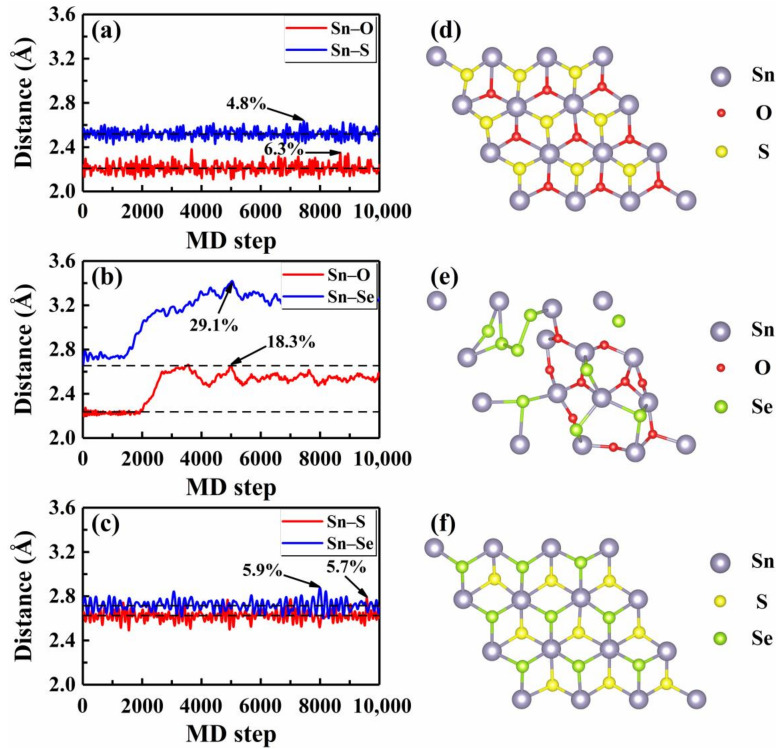
The AIMD results of the bond distances at room temperature for three Janus monolayers (**a**) SnOS, (**b**) SnOSe, and (**c**) SnSSe, where the maximum fluctuations around the equilibrium lengths are indicated. The corresponding top-views of the crystal structures at (**a**) 8680, (**b**) 5031, and (**c**) 7993 MD step are shown in (**d**), (**e**), and (**f**), respectively. The dotted lines indicate the equilibrium bond distances.

We then focused on the thermal stability of these Janus monolayers by performing AIMD simulations at finite temperatures, which can provide direct information about the structure evolutions. As an example, Figure 2a–c plot the average distances between the Sn and three nearest X and Y atoms with respect to the MD step at 300 K, where statistics were collected after 5000 running steps and the equilibrium bond lengths are indicated by the dashed lines. Generally speaking, a crystalline system tends to melt when the amplitude of atomic vibration roughly reaches 10% of the interatomic distance [44]. For the Janus SnOS, we see from Figure 2a that the maximum bond length fluctuation for the Sn–S was 4.8% (at 7450 MD step) around the equilibrium distance of 2.52 Å. In contrast, the Sn–O bond showed a relatively larger fluctuation (6.3% at the 8680 MD step). As both of them were smaller than 10%, we can safely conclude that the SnOS system is stable at room temperature. For simplicity, we considered only those bonds with larger fluctuation in the following discussions. In Figure 2c, we observe a similar picture for the Janus SnSSe, where the maximum bond length fluctuation was 5.9% at the 7993 MD step. In the case of Janus SnOSe, however, we find from Figure 2b that the bond length fluctuations were much stronger (maximum of 29.1% at 5031 MD step) which indicates that the system is unstable even at a lower temperature of 300 K. Figure 2d–f shows the snapshots of atomic structures when the monolayers SnXY exhibited the largest bond length fluctuation. Indeed, we see from Figure 2d,f that the hexagonal lattice remained almost unchanged for both the SnOS and SnSSe. In contrast, there was an obvious structural deformation for the Janus SnOSe, as observed from Figure 2e. To have a further check, additional AIMD simulations were performed at a series of temperatures from 50 K to 900 K for these three Janus structures. Detailed analysis of the bond length fluctuations (Figure S2 of the Supplementary Material) revealed that the maximum temperatures ($T_{\max}$) up to which the systems can stably exist were 825 K, 525 K, and 100 K for the Janus monolayers SnSSe, SnOS, and SnOSe, respectively. In other words, the thermal stability of these Janus monolayers was the highest for the SnSSe and lowest for the SnOSe, while the SnOS was in between.

**Figure 3:**
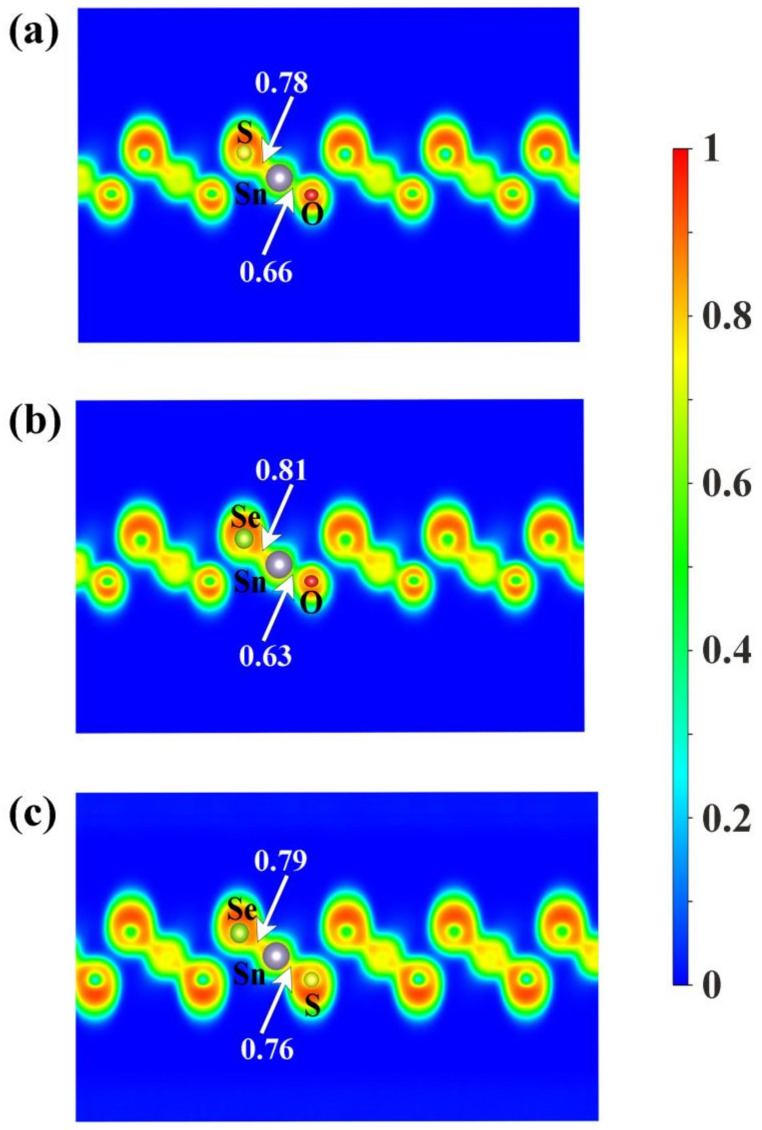
The electron localization function (ELF) maps sliced perpendicularly to the [110] direction for the three Janus monolayers (**a**) SnOS, (**b**) SnOSe, and (**c**) SnSSe. The numbers indicate the ELF values at the midpoints of the Sn–X and Sn–Y bonds.

**Figure 4:**
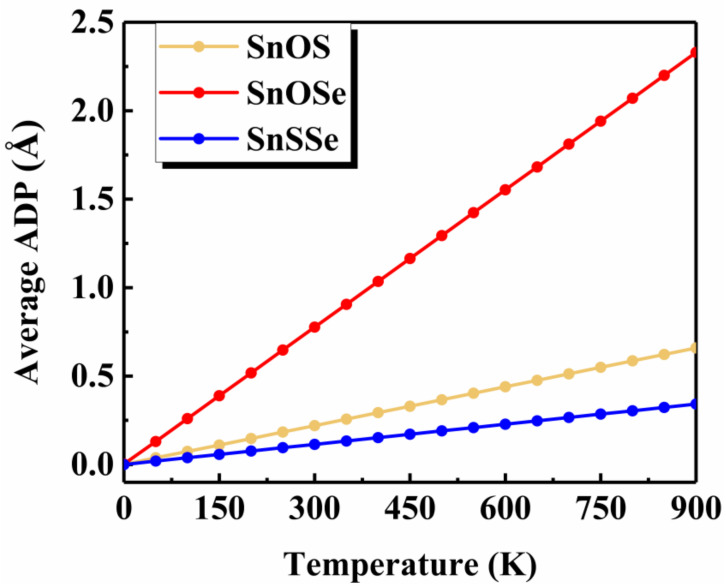
The average atomic displacement parameter (ADP) as a function of temperature for the three Janus monolayers.

To shed some light on the physical origin of obviously different thermal stability of our Janus monolayers SnXY, we first noted that the system with higher thermal stability tended to have smaller Δd (listed in Table 1). The possible reason is that larger means larger difference in the bonding strength of the Sn–X and Sn–Y so that there exists a strong vibrational mismatch at a finite temperature [45]. However, such an observation may not be applicable for other Janus systems. For example, the Δd in the PdSeTe was previously found to be 0.07 Å, which is very close to that of PdSSe (0.08 Å) with apparently higher thermal stability [39]. It is therefore necessary to figure out other essential factors governing the thermal stability of Janus SnXY. Figure 3 shows the ELF maps [46] sliced perpendicularly to the [110] direction for the three Janus structures. The value of ELF ranges from 0 to 1 and can be used to understand the bonding characteristics of a given system. If the value is close to 0, the corresponding region has a very small electron density. In contrast, the value of 1 means the complete localization of electrons [47]. As can be seen from Figure 3, all of the three Janus monolayers exhibited obvious covalent bonding nature. If we focus on the difference in the ELF of Sn–X and Sn–Y bonds ($\Delta_{\mathrm{ELF}}$), it is interesting to find that the most stable SnSSe exhibited the smallest $\Delta_{\mathrm{ELF}}$ of 0.03 while the least stable SnOSe had the largest $\Delta_{\mathrm{ELF}}$ of 0.18. For the intermediate SnOS, the $\Delta_{\mathrm{ELF}}$ was just located in between (0.12). This is reasonable since larger $\Delta_{\mathrm{ELF}}$ suggests obviously different bonding strength, which leads to stronger vibrational mismatch as discussed above. To go further, we plotted in Figure 4 the average ADP as a function of temperature for the Janus monolayers SnXY. Note that ADP measures the root-mean-squared displacement of an atom around its equilibrium position as defined by $ADP\;\;=\;\;\sqrt{\langle u_{x}^{2}\rangle +\langle u_{y}^{2}\rangle +\langle u_{z}^{2}\rangle }\;\;$, where $u_{x},u_{y},u_{z}\;$ are the displacements along the x, y, z directions, respectively. As mentioned above, the ADP can be readily calculated from the second-order IFCs rather than time-consuming AIMD simulations at various temperatures. It is obvious from Figure 4 that the ADP increased with the temperature, which was much faster for the SnOSe and obviously slower for the SnSSe, while the SnOS was in between. At a given temperature, the order of ADP was consistent with that of the thermal stability obtained by explicit AIMD simulations. That is, the higher the stability, the smaller the ADP. The fact is that a large ADP means stronger atom vibrations around the equilibrium position and the structure tends to be deformed/destroyed easily at elevated temperatures [48,49,50].

Up to now, we have demonstrated that the Janus SnXY with higher thermal stability tends to exhibit smaller Δd, as well as smaller $\Delta_{\mathrm{ELF}}$, and smaller ADP. In principle, all of them are closely related to the anharmonicity of the system, which can be characterized by the so-called Grüneisen parameter (γ) [51]. A Janus monolayer with a stronger anharmonicity (larger γ) would have larger Δd and $\Delta_{\mathrm{ELF}}$, which may lead to larger structural deformation reflected by larger ADP even at a lower temperature. Indeed, we found that the calculated γ values were respectively 0.39, 0.52, and 1.17 for the Janus SnSSe, SnOS, and SnOSe, which is consistent with the order of their thermal stability.

Although we have achieved a better understanding of the distinct thermal stability of the three Janus systems, it is time-consuming to identify their $T_{\max}$ by performing AIMD simulations at a series of temperatures. Therefore, such an approach cannot be directly used to quickly evaluate the thermal stability for a wide variety of possible Janus and similar structures. As discussed above, the SnXY structure with a larger ADP value tended to be unstable even at a lower temperature. Considering the fact that ADP can be readily obtained from the second-order IFCs [41], we propose an effective δ ratio to quickly predict the $T_{\max}$. Specifically, $\delta =\frac{\langle ADP\rangle }{a}$ where 〈ADP〉 is the average ADP and a is the lattice constant of the system. Table 2 summarizes the calculated δ values at a series of temperatures from 50 K to 900 K for the three Janus structures. It is obvious that δ increased with the temperature, and the critical values are marked which corresponds to the $T_{\max}$ discussed above. For the Janus SnSSe, SnOS, and SnOSe, the critical δ ratios were 0.08, 0.11, and 0.07, respectively. As a rule of thumb, the average of them (~0.09) was adopted to quickly predict the $T_{\max}$ of Janus and related structures. In principle, one can also take other similar systems to calculate the critical δ value, which remained ~0.09 around the $T_{\max}$ determined by explicit AIMD simulations according to our additional checks. The physical reason is that such a δ value corresponds to the upper limit of bond length fluctuation (~10%) in the AIMD simulations when the investigated systems remain thermally stable.

**Figure 5:**
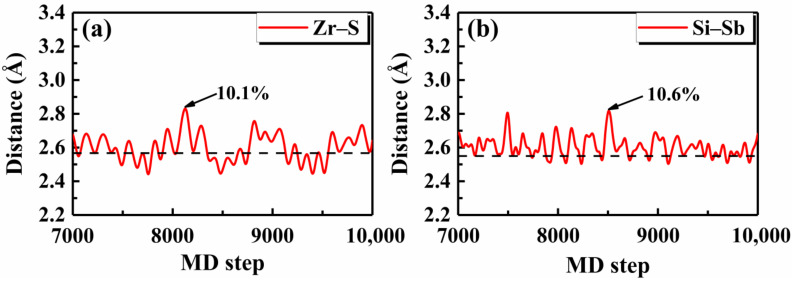
The AIMD results of the bond distances for the Janus monolayers (**a**) ZrSTe at 300 K, and (**b**) Si_2_PSb at 350 K, where the equilibrium lengths are indicated by the dashed lines and the maximum bond length fluctuations are marked.

To check the validity of such a simple rule, we first investigated the thermal stability of Janus monolayer ZrSTe, which was recently demonstrated to be dynamically stable [52]. Around the critical value of ~0.09, the $T_{\max}$ was predicted to be 225 K, as indicated in Table 3. Figure 5a shows the AIMD result for the Zr–S bond in the Janus ZrSTe and we see that the maximum fluctuation around the equilibrium bond length was 10.1% at 300 K, which is essentially close to the predicted $T_{\max}$ of 225 K considering the statistical noise of molecular dynamics simulations. Unlike the Janus ZrSTe which can be viewed as derived from the TMD structure of ZrS_2_ or ZrTe_2_, Touski et al. [53] proposed a new Janus monolayer Si_2_PSb with good dynamical stability. By calculating the corresponding δ ratio, we quickly predicted that the Si_2_PSb would be thermally stable up to 350 K (Table 3). Indeed, the AIMD result for the Si–Sb bond in the Janus Si_2_PSb showed that the maximum bond length fluctuation was 10.6% around the equilibrium distance at 350 K (Figure 5b).

It is interesting to find that our proposed δ ratio can be also used to evaluate the thermal stability of TMD monolayers, such as SnX_2_ (X = O, S, Se). As indicated in Table 3, the δ value approached 0.09 at 450 K, 600 K, and 425 K for the SnO_2_, SnS_2_, and SnSe_2_, respectively. Taking the SnSe_2_ as an example, we plotted the AIMD result in Figure S3 of the Supplementary Material. We see that the maximum fluctuation of the bond length (Sn–Se) was ~10.6% at 437 K, which can be taken as the upper temperature for the existence of a stable SnSe_2_ and was very close to our predicted $T_{\max}$ of 425 K. For the monolayers SnO_2_ and SnS_2_, the predicted values of $T_{\max}$ were slightly lower than those obtained by explicit AIMD simulations (respectively, 500 K and 675 K, see Figure S3 of the Supplementary Material). In addition, recent theoretical work found that the TMD monolayer HfSe_2_ is thermally stable at 600 K [54], which is identical to the $T_{\max}$ value predicted by our δ ratio (see Table 3). All these findings confirmed the reliability and transferability of our proposed simple rule. Compared with the time-consuming AIMD simulations at a series of temperatures, the present approach only relies on the average ADP that can be readily obtained from the temperature-independent second-order IFCs [41], enabling a much faster prediction of $T_{\max}$ up to which the systems can be thermally stable.

## 4. Summary

In conclusion, we demonstrated by AIMD simulations that the Janus monolayer SnXY (X, Y = O, S, Se) with higher thermal stability exhibited lower anharmonicity, which is consistent with the smaller difference in both the bond length and the ELF of Sn–X and Sn–Y, as well as smaller ADP. By leveraging the ratio between the average ADP and the lattice constant, we proposed a simple rule to quickly and efficiently predict the upper temperatures at which the Janus monolayers remain thermally stable, without resorting to the time-consuming AIMD simulations. With the emergence of various possible 2D Janus and similar structures, it is expected that our approach could be extendable to predictive discoveries of systems with desired thermal stability.

## Figures and Tables

**Table 1:** The lattice constant (a), the layer thickness (h), the bond lengths ($d_{\mathrm{Sn}–X}$, $d_{\mathrm{Sn}–Y}$) and their difference (Δd) of three typical Janus monolayers.

Lattice Parameters	SnOS	SnOSe	SnSSe
a (Å)	3.46	3.54	3.78
h (Å)	2.48	2.60	3.07
$d_{\mathrm{Sn}–O}\;(Å)$	2.21	2.24	/
$d_{\mathrm{Sn}–S}\;(Å)$	2.52	/	2.63
$d_{\mathrm{Sn}–\mathrm{Se}}\;(Å)$	/	2.65	2.72
Δd (Å)	0.31	0.41	0.09

**Table 2:** The summary of δ values for the three Janus monolayers SnXY at different temperatures, where the critical values at Tmax are shown in bold.

Temperature	SnOS	SnOSe	SnSSe
100 K	0.02	**0.07**	0.01
200 K	0.04	0.15	0.02
300 K	0.06	0.22	0.03
400 K	0.08	0.29	0.04
525 K	**0.11**	0.38	0.05
600 K	0.13	0.44	0.06
700 K	0.15	0.51	0.07
825 K	0.17	0.60	**0.08**
900 K	0.19	0.66	0.09

**Table 3:** The summary of δ values at different temperatures for several Janus monolayers and similar structures, where δ=0.09 are shown in bold.

Temperature	ZrSTe	Si_2_PSb	SnO_2_	SnS_2_	SnSe_2_	HfSe_2_
100 K	0.04	0.03	0.02	0.02	0.02	0.01
225 K	**0.09**	0.06	0.04	0.03	0.04	0.03
300 K	0.12	0.08	0.06	0.05	0.07	0.04
350 K	0.14	**0.09**	0.07	0.05	0.08	0.05
425 K	0.17	0.11	0.08	0.06	**0.09**	0.06
450 K	0.18	0.12	**0.09**	0.07	0.10	0.07
525 K	0.21	0.14	0.11	0.08	0.11	0.08
600 K	0.24	0.16	0.12	**0.09**	0.13	**0.09**
700 K	0.28	0.19	0.14	0.11	0.15	0.10

## Data Availability

The data presented in this study are available on request from the corresponding author.

## Supplementary Materials

The following supporting information can be downloaded at: https://www.mdpi.com/article/10.3390/nano12010101/s1, Figure S1: The phonon dispersion relations of Janus monolayers (a) SnOS, (b) SnOSe and (c) SnSSe; Figure S2: The AIMD results of the bond distances for the Janus monolayers (a) SnOS at 525 K, (b) SnOSe at 100 K, and (c) SnSSe at 825 K. For each case, only that with larger fluctuation around the equilibrium length is shown; Figure S3: The AIMD results of the bond distances for the monolayers (a) SnSe_2_ at 437 K, (b) SnO_2_ at 500 K, and (c) SnS_2_ at 675 K, where the maximum fluctuations around the equilibrium lengths are shown.
