# Supplementary material for: The Thermal Stability of Janus Monolayers SnXY (X, Y = O, S, Se): Ab-Initio Molecular Dynamics and Beyond
Source: Nanomaterials (Basel). 2021 Dec 29;12(1):101. doi: 10.3390/nano12010101 (PMC8746883; doi:10.3390/nano12010101)
Supplement: Supplementary file 1 [file nanomaterials-12-00101-s001.zip › nanomaterials-1519928-supplementary.pdf]

nanomaterials

Supplementary Material

The Thermal Stability of Janus Monolayers SnXY (X, Y = O, S, Se): Ab-Initio Molecular Dynamics and Beyond

Yufeng Luo, Shihao Han, Rui Hu, Hongmei Yuan, Wenyan Jiao and Huijun Liu *

Key Laboratory of Artificial Micro- and Nano-Structures of Ministry of Education and School of Physics and Technology, Wuhan University, Wuhan 430072, China;
luoyuf_0902@whu.edu.cn (Y.L.); hansh123@whu.edu.cn (S.H.); ruihu16@whu.edu.cn (R.H.);
hmyuan@whu.edu.cn (H.Y.); jiaowy@whu.edu.cn (W.J.)
* Correspondence: phlhj@whu.edu.cn

1. The phonon dispersion relations of Janus monolayers SnXY (X, Y = O, S, Se)

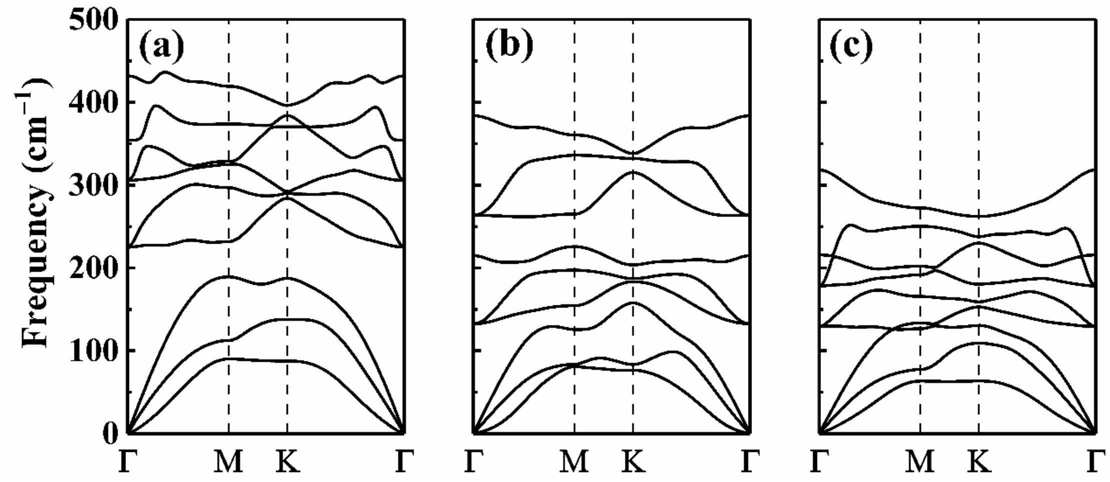

Figure S1. The phonon dispersion relations of Janus monolayers (**a**) SnOS, (**b**) SnOSe and (**c**) SnSSe.

2. The *ab-initio* molecular dynamics (AIMD) simulations for the Janus monolayers SnXY (X, Y = O, S, Se)

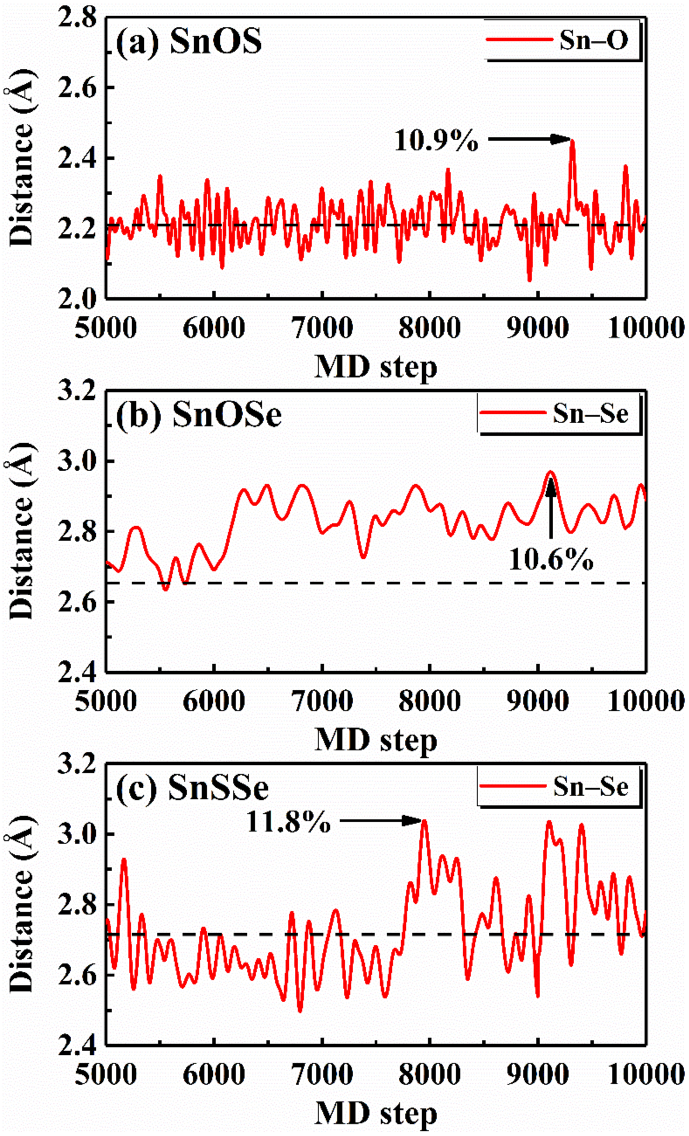

Figure S2. The AIMD results of the bond distances for the Janus monolayers (**a**) SnOS at 525 K, (**b**) SnOSe at 100 K, and (**c**) SnSSe at 825 K. For each case, only that with larger fluctuation around the equilibrium length is shown.

3. The AIMD simulations for the TMD monolayers SnX_2 (X = O, S, Se)

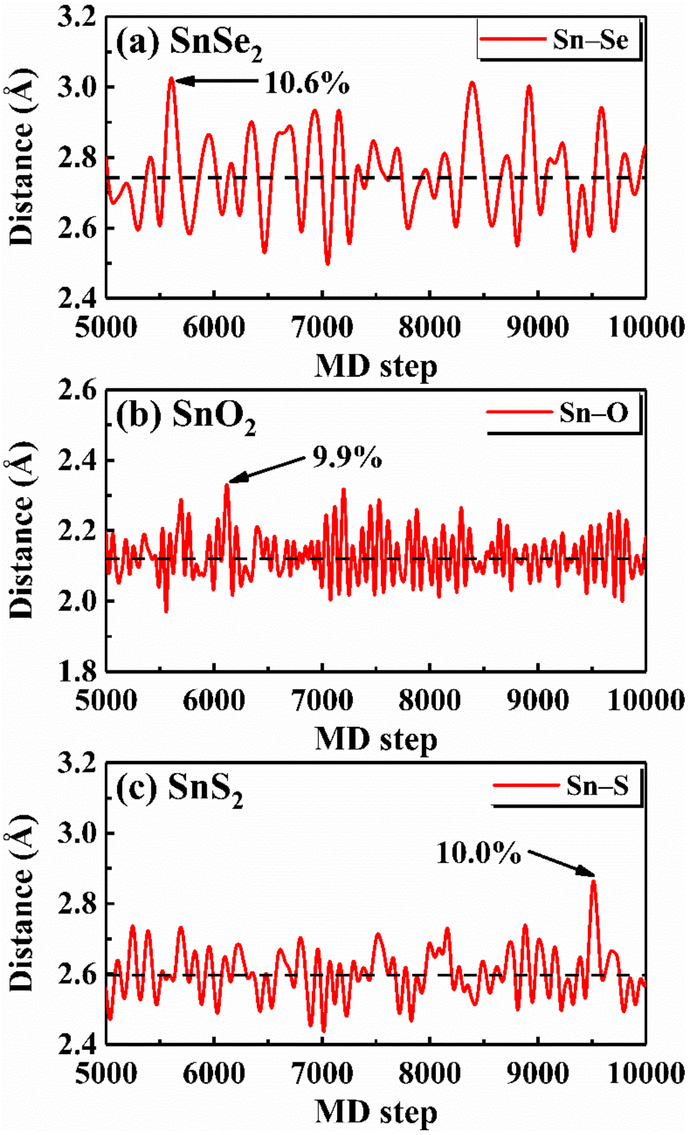

Figure S3. The AIMD results of the bond distances for the monolayers (**a**) $SnSe_2$ at 437 K, (**b**) SnO_2 at 500 K, and (**c**) SnS_2 at 675 K, where the maximum fluctuations around the equilibrium lengths are shown.
